# Health effects of nutrients and environmental pollutants in Baltic herring and salmon: a quantitative benefit-risk assessment

**DOI:** 10.1186/s12889-019-8094-1

**Published:** 2020-01-15

**Authors:** Jouni T. Tuomisto, Arja Asikainen, Päivi Meriläinen, Päivi Haapasaari

**Affiliations:** 1 Finnish Institute for Health and Welfare, Kuopio, Finland; 2 University of Helsinki, Helsinki, Finland

**Keywords:** Fish consumption, Dioxins, Methylmercury, Benefit-risk assessment, Health impact, Sperm concentration, Baltic Sea, Knowledge crystal, Food recommendation, European food safety authority EFSA

## Abstract

**Background:**

Health risks linked with dioxin in fish remain a complex policy issue. Fatty Baltic fish contain persistent pollutants, but they are otherwise healthy food. We studied the health benefits and risks associated with Baltic herring and salmon in four countries to identify critical uncertainties and to facilitate an evidence-based discussion.

**Methods:**

We performed an online survey investigating consumers’ fish consumption and its motivation in Denmark, Estonia, Finland, and Sweden. Dioxin and methylmercury concentrations were estimated based on Finnish studies. Exposure-response functions for several health endpoints were evaluated and quantified based on the scientific literature. We also quantified the infertility risk of men based on a recent European risk assessment estimating childhood dioxin exposure and its effect on sperm concentration later in life.

**Results:**

Baltic herring and salmon contain omega-3 fatty acids and vitamin D, and the beneficial impact of these fishes on cardiovascular diseases, mortality, and the risk of depression and cancer clearly outweighs risks of dioxins and methylmercury in people older than 45 years of age and in young men. Young women may expose their children to pollutants during pregnancy and breast feeding. This study suggests that even in this critical subgroup, the risks are small and the health benefits are greater than or at least similar to the health risks. Value of information analysis demonstrated that the remaining scientific uncertainties are not large. In contrast, there are several critical uncertainties that are inherently value judgements, such as whether exceeding the tolerable weekly intake is an adverse outcome as such; and whether or not subgroup-specific restrictions are problematic.

**Conclusions:**

The potential health risks attributable to dioxins in Baltic fish have more than halved in the past 10 years. The new risk assessment issued by the European Food Safety Authority clearly increases the fraction of the population exceeding the tolerable dioxin intake, but nonetheless, quantitative estimates of net health impacts change only marginally. Increased use of small herring (which have less pollutants) is a no-regret option. A more relevant value-based policy discussion rather than research is needed to clarify official recommendations related to dioxins in fish.

## Background

Dioxins (polychlorinated dibenzo-*p*-dioxins and furans) and polychlorinated biphenyls (PCBs) are persistent environmental pollutants that are found at relatively high concentrations in fish [1]. Fatty Baltic fish (such as Baltic herring, salmon, trout and lamprey) biomagnify dioxins and PCBs in the food chain; e.g. they constitute the largest exposure source of these compounds in the Finnish population [2]. These fish species often exceed the EU limits for dioxins and PCBs [3], but Finland and Sweden have a permanent derogation to sell these fish species on their national markets; Latvia has a derogation for salmon [4]. In contrast, Estonia deals with dioxins by selecting small Baltic herring with lower concentrations of pollutants for human consumption [5].

The EU has had a long-term objective of reducing human exposure to these pollutants. Emission standards for industry have become stricter during recent decades, and also concentration limits for food and feed have eliminated the most badly contaminated items from the market. Although average exposure has decreased to a fraction of previous values, there are still concerns about the health effects of dioxins, especially related to fatty fish from the Baltic Sea.

The European Commission therefore asked the European Food Safety Authority (EFSA) to perform a risk assessment and derive an updated tolerable weekly intake (TWI) for dioxins and dioxin-like PCBs. The TWI was recently published, and it is seven times lower (2 pg/kg/week) than the previous value (14 pg/kg/week) [6].

Although previous benefit-risk assessments have been published about Baltic fish [7], there are no studies that would have compared several countries and examined the reasons and motivations for fish eating (or fish avoidance).

The BONUS GOHERR project (2015–2018) evaluated the particular question about dioxins in Baltic salmon and herring and performed a health benefit-risk assessment, which is reported here. The project also studied biological, socio-economic, cultural, and food security aspects of the dioxin problem associated with the Baltic salmon and herring fisheries and the governance of the dioxin problem.

## Methods

### Modelling

The overall aim of the study was to estimate health risks and benefits of important compounds (dioxins, dioxin-like PCBs, methylmercury, omega-3 fatty acids [consisting of eicosapentaenic acid EPA and docosahexaenic acid DHA], and vitamin D) found in Baltic herring and salmon in the current situation. The assessment model was implemented in an open and modular way at Opasnet web-workspace (en.opasnet.org). In practice, this means that all the data and codes used for different parts, or modules, of the model are located on different pages at Opasnet. These pages are called knowledge crystals, as their structure and workflow follow certain rules (Tuomisto et al. 2019, forthcoming). In this section, we provide an overview of the model and describe the input data and assumptions used; the Results section consists of the results obtained with the model. Links to the module pages and all details can be found in the assessment page [8]. The whole model with data and codes is available on the page and also at Open Science Framework research data repository [9].

The benefit-risk assessment was based on a modular Monte Carlo simulation model, which had a hierarchical Bayesian module for estimating dioxin concentrations. The different modules are briefly described below, with references and links to further material.

The input distributions were derived either directly from data or from scientific publications. If no published information was available (e.g. as was the case with disability weights for non-typical endpoints such as tolerable weekly intakes or infertility), we used author judgement and wide uncertainty bounds (these judgements are described later in the text). The model was run with 3000 iterations using R statistical software (version 3.5.3, https://cran.r-project.org).

### Consumption survey

**Fig. 1:**
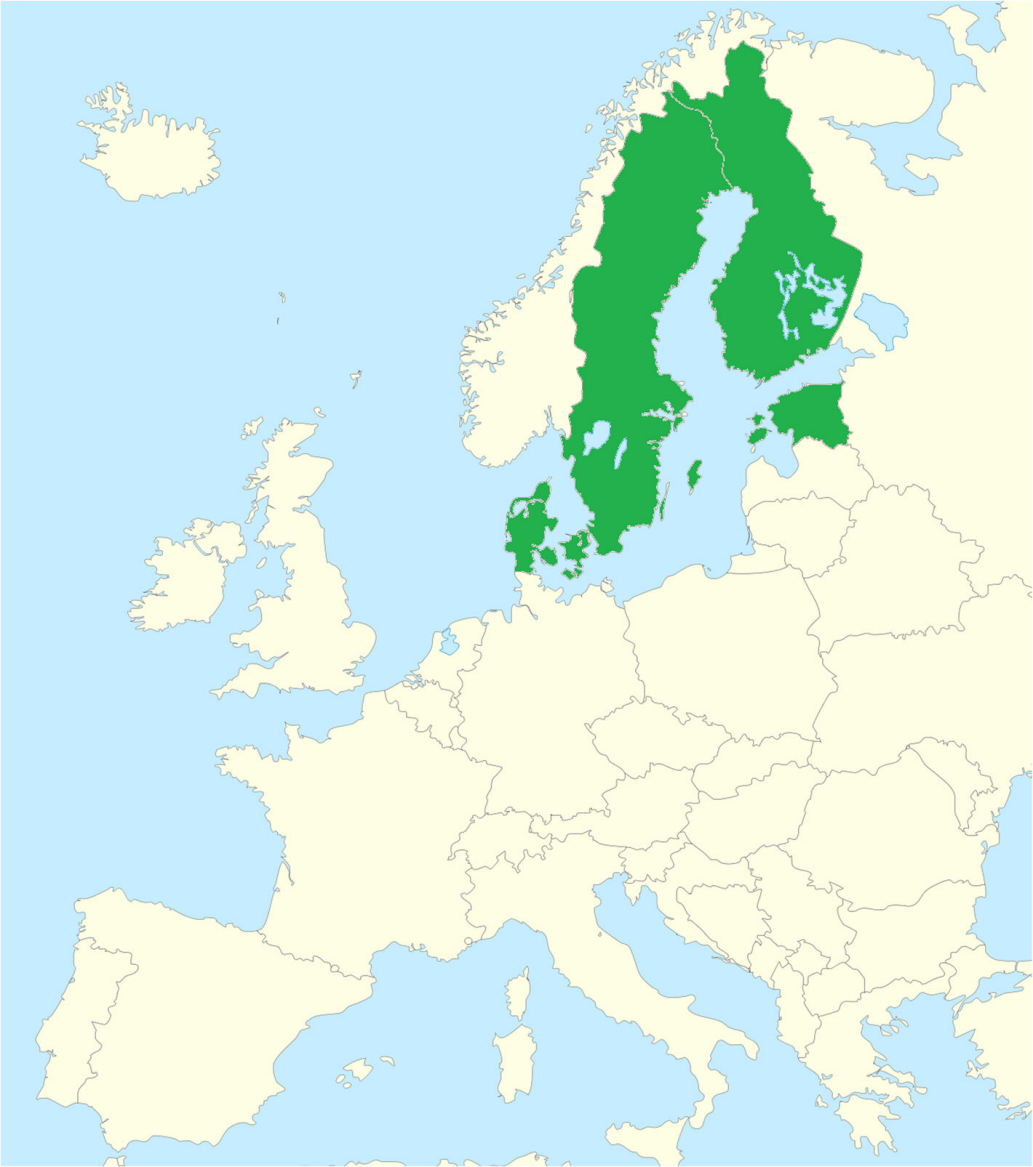
Countries where the BONUS GOHERR consumption survey was performed. Modified from https://commons.wikimedia.org/wiki/File:Blank_map_of_Europe_-_Atelier_graphique_colors_with_Kosovo.svg

The data used were from an internet-based survey that was conducted at the end of 2016. The survey focused on consumers’ eating habits of Baltic herring and salmon in four Baltic Sea countries: Denmark, Estonia, Finland, and Sweden (Fig. 1). The questionnaire was designed and the results analysed by the authors, but the survey was administered by a professional market research company Taloustutkimus oy, which has an established internet panel since 1997. The survey company recruited over 500 consumers from each country (total 2117) to respond to the survey questionnaire, which is above the required sample size to allow generalization of the results to each case country studied (with a 95% confidence level and 5% margin of error) [10]. The survey was targeted to the adult population, i.e. 18 years or older.

The survey questionnaire comprised 32 questions, including sociodemographic questions as well as questions relating to the frequency and amount of fish consumption in general, and to Baltic herring and salmon in particular. There were also questions about reasons to eat or to not to eat those species, and policies that may affect the amount eaten. The questionnaire was translated into the national language of each case study country (Finnish, Swedish, Estonian and Danish). The country and gender of the respondents were provided directly by the internet panel and were therefore not included in the questionnaire.

Only those respondents who reported fish consumption in general were asked follow-up questions about herring and salmon consumption, and are included in the analysis presented in this paper. As the survey focused specifically on the consumption of herring and salmon originating from the Baltic Sea, a distinction had to be made in the questionnaire between Baltic herring and herring originating from elsewhere, e.g. the North Sea or North Atlantic, as well as between the salmonids (Baltic and Norwegian salmon, farmed salmon, rainbow trout). With regard to herring consumption, those respondents that reported eating some type of herring were asked explicitly whether they consumed Baltic herring. Concerning Baltic salmon, the respondents were asked to choose the fish that they consumed from a list of salmonids. The survey was designed and conducted for the purposes of this study and another study investigating consumers’ perception and consumption of fish. The latter study [11] was published first, and it contains a more detailed description of the study methods, including the questionnaire.

Individual long-term fish consumption (in kilograms per year) was estimated from the questions about consumption frequency and amount. Consumption distributions were produced for subgroups defined by country, gender, and age by random sampling (with replacement) of the individual estimates. People’s reactions to changes in policies or fish market (e.g. what if fish consumption is recommended or restricted; what if the availability and usability of these species improve; what if the price of fish changes) were predicted based on their answers. These decision scenarios were used to alter the business-as-usual scenario and compare results between scenarios.

In addition, a few technical scenarios were developed: what if nobody ate more fish than ca. 1 kg per year; and what if fish are considered as a primary versus a secondary source of nutrients. The latter scenario is important if dose-responses are non-linear, as is the case with vitamin D. In such a case, the incremental health benefits of a primary source may differ from those of a secondary source.

The data analysis was conducted using R software (version 3.5.3, http://cran.r-project.org). Because the survey was conducted on an internet panel rather than on a random sample from the general population, the respondents may not be fully representative of the actual population distributions in the countries. Therefore, the respondents were weighted based on actual age, gender, and region distributions of each country to produce population representative results.

To support transparency, the anonymised data and all the results will be available online: http://en.opasnet.org/w/Goherr:_Fish_consumption_study

### Concentrations

Fish-size-specific PCDD/F and dioxin-like PCB concentration distributions for each fish species and country were estimated based on EU Fish II study [12]. The results were based on pooled and individual fish samples (98 Baltic herring and 9 salmon samples) and analysed for 17 dioxin and 37 PCB congeners. A hierarchical Bayesian module was developed with the JAGS package of R software. The model assumed ca. 7% annual decrease in dioxin concentrations, based on long time trends measured in Finland. The fish samples were caught between 2009 and 2010.

The concentrations in Baltic herring were noted to be highly sensitive to fish size, whereas size-dependency was much weaker in salmon. Herring sizes in different scenarios came from a fish growth model developed in BONUS GOHERR project [13].

The fish samples came mostly from the Bothnian Sea, which is an important catchment area for the Finnish and Swedish fisheries. The concentration distributions for the studied countries were derived from the concentration model results by scaling them with the average concentration on a catch area of interest relative to the average from the Bothnian Sea. The Danish catch areas were assumed to be Baltic west of Bornholm whereas the Estonian catch area was the Gulf of Finland. The Swedish catch areas for herring and salmon were assumed to be the Baltic Main Basin and the Bothnian Sea and Bay, respectively. The area selection was based on landing statistics provided by the International Council for the Exploration of the Sea (ICES) [14, 15].

Dioxin and PCB concentrations were weighted and summed up to toxic equivalency quantities (TEQ) by using WHO 2005 toxic equivalency factors (TEF) [16]. Levels of fatty acids and vitamin D in Baltic herring were based on measurement data obtained from the Finnish Food Safety Authority, and those in salmon are based on the Fineli food database [17]. Methylmercury concentrations were based on the Kerty database [18].

### Exposures

Exposures to pollutants and nutrients were simply products of consumption amounts as assessed from the survey and concentrations in the consumed fish, with possibly an uncertain background intake from other sources. One exception to this was the exposure by infants to dioxins and methylmercury during pregnancy and breast-feeding; these were derived from the mother’s exposure using toxicokinetic models.

Infant’s exposure during pregnancy and breast-feeding was estimated with this equation:

$$ {C}_{s,i}=\frac{I_{a,m}\ast {t}_{1/2,m}\ast {f}_m\ast FE}{\ln 2\ast B{F}_i}, $$

where C_s,i_ = serum (s) concentration of dioxins in the infant (i) in pg/g fat; I_a,m_ = average daily intake of dioxins of the mother (m) in absolute (a) amounts pg/day; t_1/2,m_ = the half-life of dioxins in the mother (2737.5 d = 7.5 a); f_m_ = fraction of ingested dioxins actually absorbed from the gut of the mother (0.80); FE = fraction of mother’s dioxin load that is transported to the infant during breast feeding (0.25); BF = body fat amount in the infant (into which the dioxins are evenly distributed) during the period when tooth and testis are sensitive to defects and the exposure is at its highest (ca. 6 months of age) (1 kg) [19].

### Exposure-responses

Several benefits and risks were assessed (Table 1). We tried to choose impacts that are arguably large enough that could affect the benefit-risk balance. The effects of omega-3 fatty acids on coronary heart disease, mortality and child’s intelligence, as well as the effects of vitamin D on vitamin deficiency belong to this category. In contrast, there are several endpoints that have been linked to fish or omega-3 intake, but they were not included because current evidence is controversial: diabetes [32], prostate cancer [33], asthma [34], and stroke [24].

**Table 1:** Exposure-response functions used in the assessment

Exposure agent	Response	Esposure-response unit	Exposure-response function mean (95% confidence interval)	References and notes
TEQ (intake through placenta and mother’s milk)	male infertility due to sperm concentration decrease	pg /g in boy’s body fat	linear; slope 0.00006 (− 0.000019, 0.00014)	Based on EFSA TWI assessment [6]. Mother’s exposure must be converted to child’s exposure (measured as pg /g fat) [20]
TEQ (intake through placenta and mother’s milk)	developmental tooth defects	log (pg /g) in child’s body fat	linear; slope 0.0014 (0.00029, 0.0025)	epidemiological study in Finland [21]
TEQ	cancer morbidity	pg/kg/day	linear; slope 0.00051 (0.000026, 0.00097)	U.S.EPA dioxin risk assessment [22].
TEQ	tolerable weekly intake 2001	pg/kg/week	acceptable range below 14	EC Scientific Committee on Food recommendation [23]
TEQ	tolerable weekly intake 2018	pg/kg/week	acceptable range below 2	EFSA recommendation [6]
Omega-3 fatty acids	coronary heart disease mortality	mg/day	ED50: −0.17 (− 0.25, − 0.091)	Cochrane review [24]
Vitamin D	vitamin D recommendation	μg/day	acceptable range 10–100	a step function based on the daily intake recommendations for adults in Finland [25]
ALA	coronary heart disase mortality	mg/day	RR 0.95 (0.72–1.26)	after 1000 mg/d of alpha-linolenic acid intake; Cochrane review [24]
Omega-3 fatty acids	breast cancer	mg/d	RR 0.95 (95% CI 0.90, 1.00)	after 0.1 g/d of marine omega-3; a meta-analysis [26]
Fish	all-cause mortality	g /d	RR 0.88, (95%CI 0.83, 0.93)	after 60 g/d of fish; a meta-analysis [27]
Fish	depression	g/d	RR 0.83 (95% CI 0.74, 0.93)	after 35 g/d of fish; a meta-analysis [28]
Methylmercury	loss in child’s IQ points	mg/kg/day	linear; slope 6.6 (− 0.27, 14)	a synthesis of EFSA TWI estimate [29] and a previous risk assessment [30].
DHA	loss in child’s IQ points	mg/day	linear; slope − 0.0013 (− 0.0018, − 0.00081)	a previous risk assessment [31].

In addition, many studies have linked health benefits to fish consumption rather than to a specific nutrient [35]. We included depression [28], breast cancer [26], and all-cause mortality [27], because the results have been rather consistent in the meta-analyses. In summary statistics, CHD and breast cancer mortalities were subtracted from all-cause mortality to avoid double-counting.

The dioxin effect on sperm concentratio n[6] and methylmercury effect on intelligence in children [36] are the most sensitive risks of these pollutants, and they were therefore included.

In addition, we included some other dioxin effects. Tolerable weekly intakes from 2001 and 2018 were included for comparing methods of quantitative benefit-risk assessment (based on a single health aggregate, DALY) and more qualitative benefit-risk assessment (based on assessing whether a beneficial or harmful threshold is exceeded). A cancer effect was included because the news media often refers to dioxins as “the super poison causing cancer” although researchers have believed for years that it is the developmental problems rather than cancer risks that are more relevant; a quantitative assessment could give guidance to media communication. Finally, tooth defects were included because it is a sensitive dioxin endpoint, but no study has compared its magnitude to effects on sperm quality. Interestingly, some of the key papers assessing this effect had been conducted in Finnish mothers who had been exposed to dioxins, mostly from Baltic herring [2, 21, 37].

The exposure-response function of methylmercury was a synthesis of EFSA tolerable weekly intake and a linear function from Cohen et al. [30]. This was necessary because although the EFSA estimate is fairly recent, it does not quantify the magnitude of the effect if the TWI is exceeded. The function by Cohen was based on concentrations measured from mothers’ hair. A conversion from hair concentrations to daily exposures was performed according to U.S.EPA [36].

We derived the exposure-response functions for infertility and tooth defects indirectly from published results, and thus the rationale of those endpoints will be described here in more detail.

In humans, sperm concentrations have been shown to decrease permanently if boys are exposed to dioxins before they are 9 years old. This data originates from Seveso [38, 39] and a Russian children’s study [20].

EFSA recently assessed this risk from the Russian children’s study and concluded that a significant effect was seen already in the second quartile with a median PCDD/F TEQ concentration of 10.9 pg/g fat, when measured from the serum of the boys at the age of ca. 9 years’ old. Mean sperm concentration was ca. 65 (95% CI 50–80) million/ml in the lowest quartile, while in all other quartiles, the concentration was ca. 40 (95% CI 30–55) million/ml. Due to the shape of the effect, we used a non-linear exposure-response curve with half of the maximum effect (effective dose 50, ED50) occurring at a TEQ concentration of 10 pg/g fat.

However, a reduced sperm concentration as such is not an adverse health effect. It only manifests itself if the concentration is low enough to prevent conception in a reasonable time window, e.g. 5 years. According to a review, the success rate of couples who try to have a child is 65% in 6 months if the male’s sperm concentration is above 40 million/ml [40]. Below that concentration, the probability is rather proportional to the sperm concentration.

Based on this, we estimated that (assuming independent probabilities between 6-month periods), the probability of not becoming pregnant in 5 years follows this curve:

$$ P(a)={\left(1-0.65\ \left(1+\frac{-0.39c}{c+10\  pg/g}\right)\right)}^{2a}, $$

where c is the dioxin concentration in pg/g in the boy’s fat tissue and a is the follow-up time in years (in this case, 5). This curve is rather linear below a TEQ concentration 50 pg/g with slope ca. 0.00006 g/pg, meaning that for each 1 pg/g increase in the dioxin concentration in the boy’s fat tissue (or serum fat), there is an incrementally increased probability of 0.00006 that he cannot father a child even after 5 years of trying.

The exposure-response function for the tooth defect was also derived from several studies. Alaluusua and coworkers have studied dioxin exposure in small children and the development of permanent molar teeth. They have found defects in both the general population in Finland attributable to the exposures in the 1980’s [21, 37] as well as in children exposed during the Seveso accident [41].

Based on these studies, we approximated that the effect would be linearly correlated with the logarithm of the dioxin concentration in the child.

### Disease burden

**Fig. 2:**
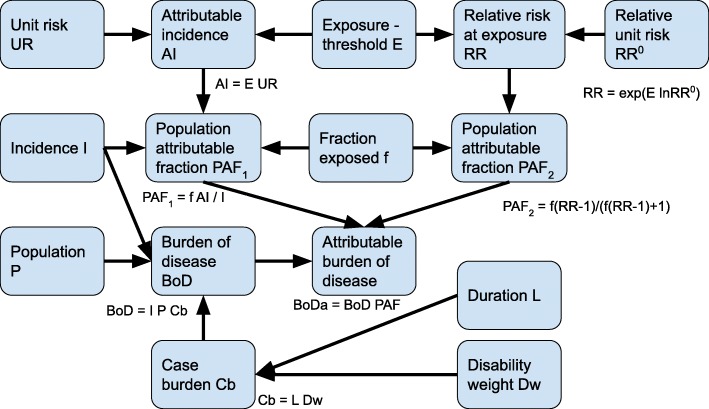
Schematic diagram of the health impact assessment model used. Each blue node is a submodel, and arrows are functional relations. The main equation of the relations is shown beside each node

Disease burden [42] was estimated in one of two alternative ways (Fig. 2): if an exposure agent affects the burden of a particular disease in relation to the background of the disease, the attributable fraction of a particular compound exposure was calculated. If the relationship was not relative to background, the attributable number of cases due to the exposure was estimated, and this was multiplied by the years under disease per case and the disability weight of the disease (Table 2.).

**Table 2:** Case burdens of different health responses. Case burden is calculated as the product of disease-specific disability weights and disease durations

Response	DALYs per case	Description
Tooth defect	0–0.12	disability weight 0.001 and duration 60 a (years) with 100% uncertainty. For comparison, IHME gives disability weight 0 for asymptomatic caries and 0.006 for mild other oral disorders with symptoms [43].
Cancer	19.7 (17.8–21.8)	based on breast cancer, from IHME [44]
Vitamin D intake	0.0001–0.01	disability weight 0.001 and duration 1 a with 100-fold log-uniform uncertainty
TWI 2001	0.0001–0.01	disability weight 0.001 and duration 1 a with 100-fold log-uniform uncertainty
TWI 2018	0.0001–0.01	disability weight 0.001 and duration 1 a with 100-fold log-uniform uncertainty
Infertility	0–5	disability weight 0.1 and duration 50 a with 100% uncertainty. See also text. Here we used a clearly higher disability weight than IHME (0.008) [43].
Child’s IQ	0.11 (95% CI 0.06–0.16)	Mild intellectual disability (IQ < 70) has disability weight 0.043 (95% CI 0.026–0.064) based on IHME [43]. This is scaled to one IQ point with duration 75 a.

$$ {\displaystyle \begin{array}{l} Bo{D}_i= BoD\ast PA{F}_i= BoD\ast {f}_i\frac{R{R}_i-1}{f_i\left(R{R}_i-1\right)+1}, or\\ {} Bo{D}_i={N}_i\ast L\ast Dw=P\ast {f}_i\ast U{R}_i\ast {E}_i\ast L\ast Dw,\end{array}} $$

where BoD is the burden caused by the disease under study, i is an exposure agent affecting the risk of the disease, PAF is a population attributable fraction, f is the fraction of the population that is exposed, RR_i_ is the relative risk that the population faces due to the studied level of exposure to the exposure agent i (as compared with a counterfactual scenario with no exposure), N_i_ is the number of disease cases attributed to exposure agent i, L is the duration of a disease incident, Dw is the disability weight of the disease (0 = perfect health, 1 = death), P is the population size, UR is the absolute unit risk, and E is the exposure to the agent.

Background disease burdens were needed for all-cause and coronary heart disease mortality, as well as breast cancer and depression; they were obtained from the Institute for Health Metrics and Evaluation (IHME) (Table 3.) [45]. The disease burden of a cancer case was based on IHME data. Furthermore, disability weights of diseases were based on their estimates, if available. Duration estimates of diseases were mostly based on the time window considered (1 year) or lifetime (in the case of permanent infertility, tooth or IQ effects due to infant exposure). We tried to be realistic with estimates but also not to underestimate the risks of fish consumption, so that potential conclusions about the safety of fish would not be unfounded.

**Table 3:** Total burden of disease of selected causes from all risk factors in the study countries [44]

Disease	1000 DALYs per year, mean (95% CI)			
	Denmark	Estonia	Finland	Sweden
Breast cancer	20 (11, 30)	3.9 (2.5, 5.6)	16 (10, 23)	30 (18, 42)
Depression	21 (18, 25)	7.6 (6.4, 8.8)	33 (27, 38)	62 (51, 73)
Heart (CHD)	84 (79, 88)	54 (47, 61)	150 (140, 160)	200 (190, 210)
Mortality	810 (780, 840)	250 (240, 270)	800 (770, 830)	1200 (1180, 1250)
Vitamin D intake	2.1 (0.11, 9)	0.5 (0.026, 2.1)	2.1 (0.11, 8.8)	3.7 (0.19, 16)

With the non-typical health effects, namely due to exceeding tolerable weekly intakes and deviation from the vitamin D recommendation, we used very wide uncertainty distributions, as it was unclear how much weight should be given to endpoints that are only indications of a potential health risk rather than actual adverse effects. A value of information analysis was performed to test the importance of these uncertainties.

Childlessness can be viewed as tragedy of life, so the disability weight could be in the order of 0.1 DALY per year permanently (50 years). However, the disability weight applies to only half of the children (boys). Therefore, we used 0.1*50*0.5 DALY/case = 2.5 DALY/case, with a rather high uncertainty (0–5 DALY/case).

Population data for each country for year 2016 was available from the Eurostat database. Data was separated for gender and age (18–45 years and > 45 years) groups [46].

### Value of information analysis

Value of information is a mathematical method that compares the difference of utility (money, DALYs or other measure of the objective) in two scenarios: that some additional information is obtained before a decision is made, or that the decision is made with the current information. This can be formulated as

$$ VOI=E\left( ma{x}_i\left(U\left({d}_i\right)\right)\right)- ma{x}_i\left(E\left(U\left({d}_i\right)\right)\right), $$

where VOI is value of information, E is expected value, U is the utility of decision d, and i is an index of decision options [47]. In this study, we also estimated the value of including or excluding an option in the decision making.

## Results

**Fig. 3:**
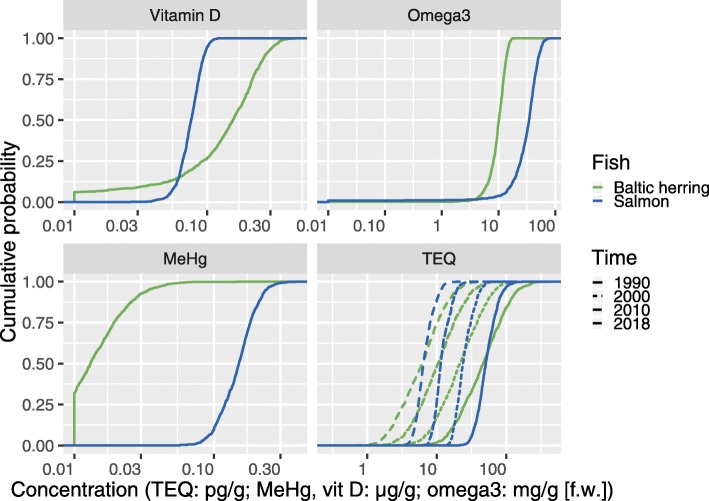
Cumulative concentration distributions of the four key exposure agents in Baltic herring and salmon. For dioxin, also the decreasing time trend since 1990 is shown. Concentrations of compounds in Baltic fish

Concentration distributions of the key exposure agents in Baltic herring and salmon are shown in Fig. 3. Baltic herring have lower concentrations than salmon for most of the exposure agents studied, but for vitamin D, the levels in salmon are lower. Dioxin concentrations have declined substantially since 1970; the trend since 1990 is shown in Fig. 3.

**Fig. 4:**
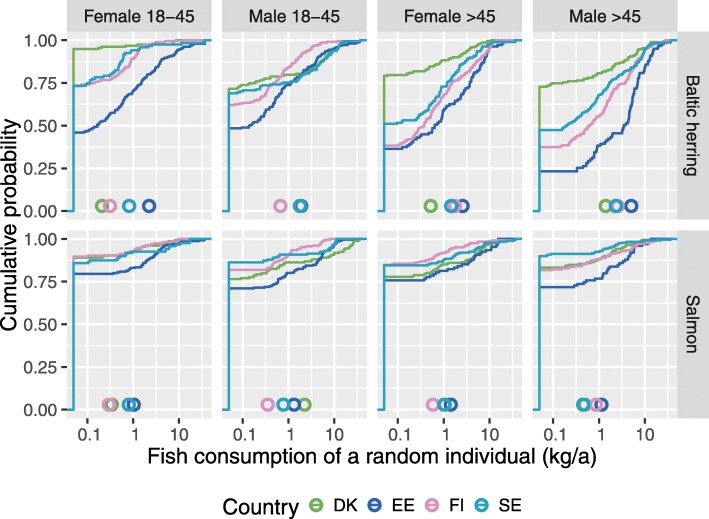
Cumulative fish consumption distributions of Baltic herring and salmon in different subgroups of the studied countries. The mean of each distribution is shown with a circle. Consumption of Baltic fish by country and subgroup

Fish consumption varies extensively between countries and population subgroups, and also within each subgroup (Fig. 4.). There is extensive individual variation (almost a hundredfold) in fish consumption within most subgroups.

Only about a quarter of people reported any wild Baltic salmon consumption. Many people also say that they do not know where their salmon comes from and whether it is salmon or rainbow trout. Herring consumption is more accurately known, although the Danes are not sure whether their herring comes from the Baltic Sea or the North Sea. For example, in Finland a typical dish name contains the word *herring* (*silakka* in Finnish), if it contains Baltic herring. Therefore the species is often known to the consumer even if the dish is not homemade. However, this is not true with Baltic salmon.

There is also extensive variation between population subgroups. Estonians eat clearly more Baltic herring and Danes eat less than individuals from the other countries. Males tend to eat more, and young people eat less than other population subgroups. These differences are rather similar in all countries, although at different levels. The fraction of people that do not eat Baltic herring at all varies remarkably between subgroups: it is only 25% in old male Estonians, while it is more than 90% in young female Danes. There is also a sizable fraction of people who eat more than 3.6 kg/year (10 g/day) of Baltic herring. This varies from a few percent in young people up to about 30% in old Estonians.

The average consumption of Baltic herring and salmon is 1.4 and 0.5 kg/a per person, respectively, in Finland according to estimates based on our survey. However, the Natural Resources Institute Finland has reported (mostly based on catch-landing statistics) that the consumptions of Baltic herring and salmon were 0.31 and 0.07 kg/a per person, respectively [48]. This implies that people tend to overestimate their long-term average consumption in general and for Baltic salmon in particular. Because of this discrepancy, we performed a sensitivity analysis where our consumption estimates were scaled to match the Finnish statistics (data not shown). The results of all variables were smaller, but the overall picture remained the same. For example, a notable fraction of population still exceeded the TWI 2018 value.

**Fig. 5:**
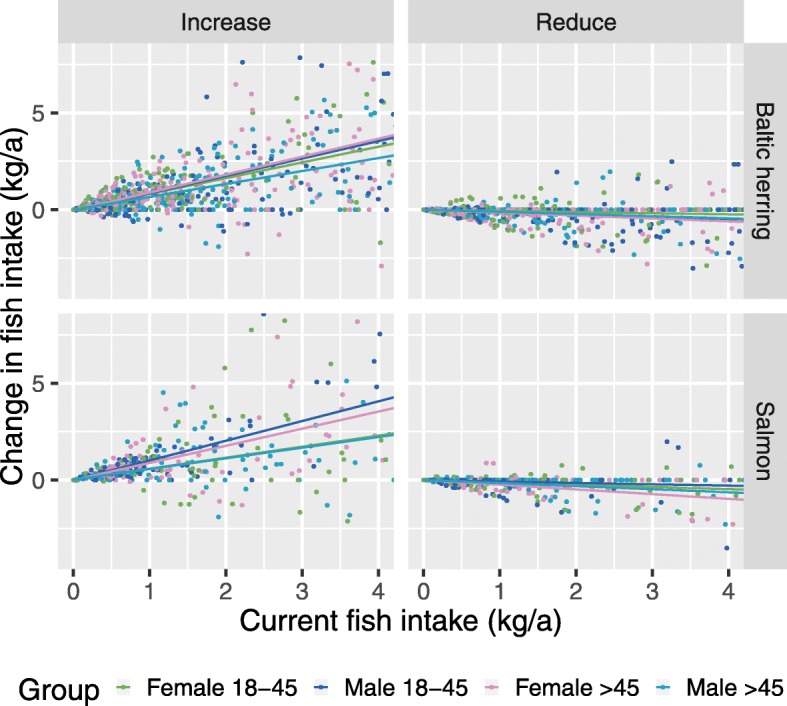
Individual change in consumption after policies to either increase or reduce fish intake. Individuals’ fish intake taking account of different consumption policies

We also asked in the questionnaire how the respondent would change his/her fish intake if an increase or decrease of fish consumption was recommended by the authorities (Fig. 5.). The outcome depends on previous consumption but not so much on the population subgroup. If an increase is recommended, a clear and systematic increase is seen in the average response. In contrast, a recommendation to reduce intake results in inconsistent effects. Some people would follow the recommendation, but almost an equal number would not, and most would not change their intake of fish. This phenomenon is seen already at current intake levels below 1.8 kg/year, which is the level consumed by most of the population.

**Fig. 6:**
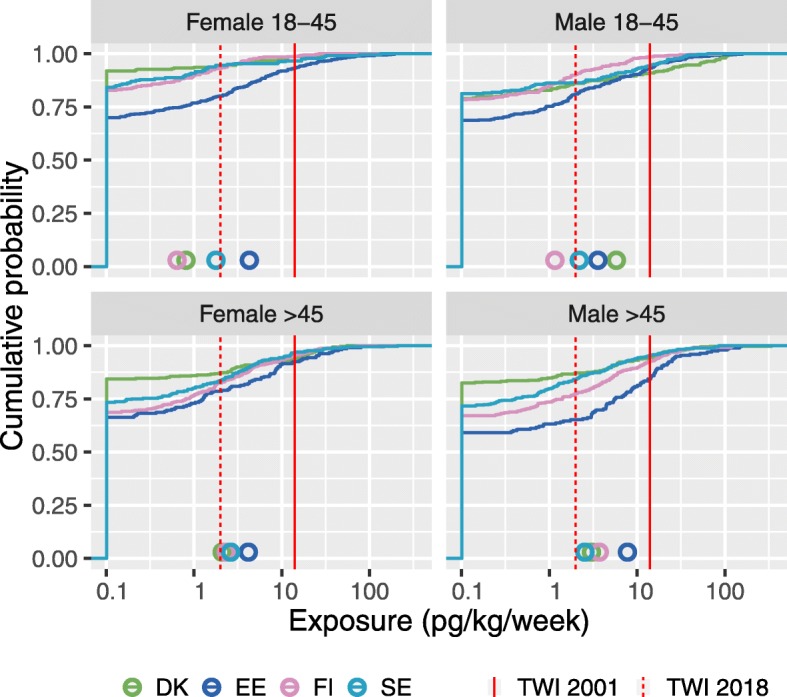
Cumulative dioxin (TEQ) exposure distributions shown by subgroup and country. The mean of each distribution is shown with a circle. Exposure to dioxins from Baltic fish

Because of the large variation in fish consumption, also the dioxin exposure from Baltic fish varies by more than a hundred-fold within the different population subgroups (Fig. 6.) and there is also extensive variation between the subgroups. In the model, many people have apparently zero exposure because dioxin sources other than Baltic fish were not included. A fraction ranging from a few percent to a quarter exceed the EC Scientific Committee on Food TWI value from 2001 [23]. The fraction becomes much higher, from 20% to up to 75%, when the new EFSA TWI value of 2 pg/kg/week from 2018 is used as the criterion [6].

**Fig. 7:**
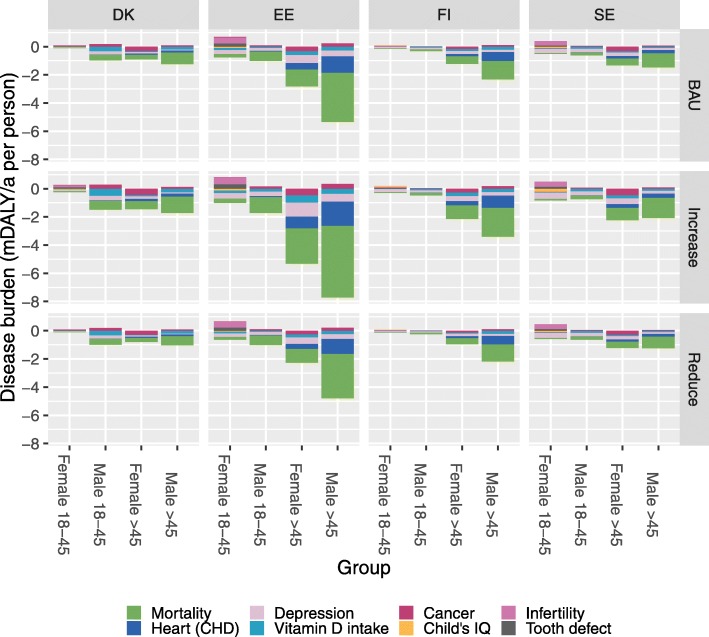
Disease burden attributable to eating Baltic fish in Denmark, Estonia, Finland, and Sweden (expected value at the individual level). Note that negative values refer to improved health. mDALY: 0.001 disability-adjusted life years, CHD: coronary heart disease, IQ: intelligence quotient. Disease burden attributable to consumption of Baltic fish by country, group, and policy

The main objective of this study was to compare health risks and benefits of Baltic fish consumption. The most dominant feature is the health benefits from all-cause mortality, ischemic heart disease, and depression (Table 4). Figure 7. shows the large variation between population subgroups. In old age groups, the benefits clearly outweigh all risks but in most population subgroups, the benefits are typically much larger than risks. In contrast, the risks and benefits in young women are both small, but at the individual level, risks are often larger according to the model.

**Table 4:** Burden of disease related to Baltic herring and salmon consumption

Response	Exposure agent	DALYs per year, mean (95% CI)			
		Denmark	Estonia	Finland	Sweden
Cancer (all)	TEQ	160 (0, 1300)	57 (0, 370)	150 (0, 1300)	260 (0, 2200)
Cancer (breast)	Omega-3	− 310 (− 3700, 0)	−55 (− 450, 0.002)	−130 (− 1400, 0)	− 330 (− 4000, 0)
Child’s IQ	DHA	−88 (− 1000, 0)	−180 (− 1300, 0)	− 58 (− 370, 0)	− 540 (− 7600, 0)
Child’s IQ	MeHg	390 (0, 1900)	170 (0, 1100)	56 (0, 270)	370 (0, 6100)
Depression	Fish	−130 (− 1200, 0)	−79 (− 430, 0)	− 170 (− 1300, 0)	− 420 (− 3000, 0)
Heart (CHD)	ALA	3.1 (− 220, 290)	− 20 (− 150, 37)	− 40 (− 560, 280)	−47 (− 880, 510)
Heart (CHD)	Omega-3	−190 (− 2600, 200)	− 120 (− 1400, 69)	−300 (− 2300, 120)	− 530 (− 7300, 820)
Infertility	TEQ	110 (0, 1100)	66 (−23, 410)	44 (0, 470)	75 (0, 460)
Mortality	Fish	−2200 (− 20,000, 0)	−1200 (− 8300, 0)	− 2800 (− 21,000, 0)	− 5600 (− 44,000, 0)
Tooth defect	TEQ	160 (0, 2400)	38 (0, 170)	11 (0, 130)	34 (0, 480)
TWI 2001	TEQ	400 (0, 6000)	87 (0, 1400)	180 (0, 2400)	460 (0, 6100)
TWI 2018	TEQ	690 (0, 7400)	310 (0, 2100)	660 (0, 5700)	1000 (0, 12,000)
Vitamin D intake	Vitamin D	−88 (− 490, 0)	−42 (− 340, 0)	− 59 (− 420, 0)	−200 (− 1800, 0)

**Fig. 8:**
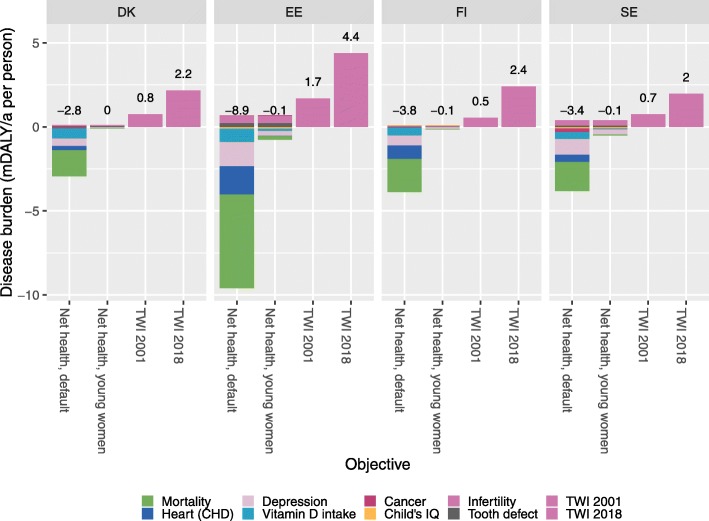
Outcome of interest using different objectives. The default objective (the main assessment of this article) focusses on the total net health effect in the whole population. The second objective focusses on young women only. Tolerable weekly intakes from 2001 and 2018 are converted to DALYs based on the number of people exceeding the guidance value. Disease burden using different objectives

Figure 8 illustrates several different objectives that could be used as a basis for decision making. The first one is using a net health effect, estimated similarly to the quantitative benefit-risk assessment performed here. The second objective focuses only on young women as the target group facing the risks, and ignores the impact on others. The third and fourth objectives were to try to avoid exceeding tolerable weekly intake values from 2001 and 2018, respectively.

When the whole population is considered (left bar), the net health objective recommends increasing rather than decreasing Baltic fish consumption in every country, while approaches based on TWI suggest that reducing fish consumption is a better option. If only the target population of young women is considered (second-left bar), all impact values are close to zero, but the net health impact may sometimes point to a slightly larger risk than benefit; the situation is ambiguous but the stakes are not high.

In general, health impacts are much smaller in young age groups, and in young women, the critical issues to be considered are the effects on their children’s intelligence quotient (IQ), tooth defects, and sperm concentration, not the health impacts on the woman herself. These risks emerge due to dioxin and methylmercury exposures during pregnancy and breast feeding. The child’s own diet during his/her early years may also have an impact, although the exposure then is typically much lower. These risks are in the same range as the health benefits, and the overall balance depends mostly on the disability weights of distinct outcomes and other value judgements such as whether Baltic fish is considered as a primary source of nutrients.

Based on our survey, a policy of recommending increased consumption seems to be somewhat beneficial, while a recommended consumption reduction is indistinguishable from the business-as-usual scenario. In contrast, policy decisions and actions to reduce dioxin emissions and consequently exposures have been very effective during the last 40 years (see Fig. 3 for concentration trends).

**Fig. 9:**
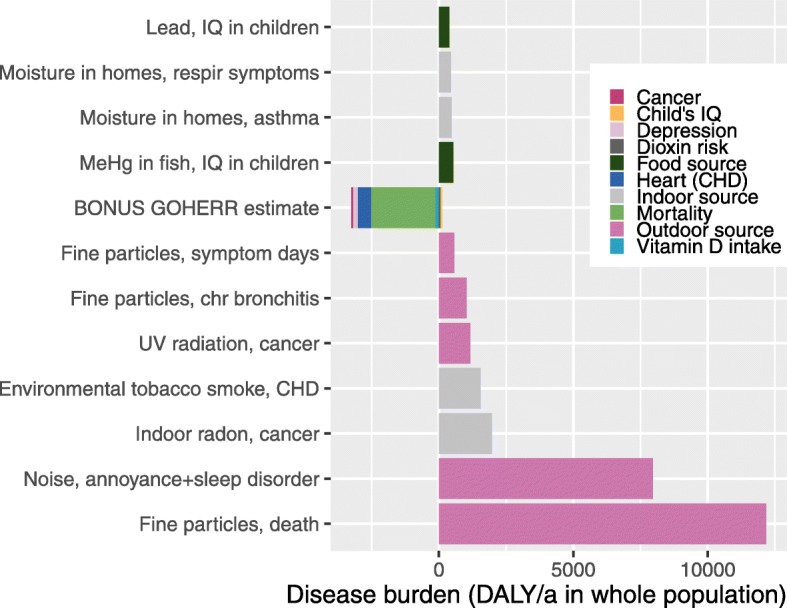
Burden of disease of the most important environmental health factors in Finland. BONUS GOHERR results are from this study, other results are from a previous publication [49]. Environmental disease burden in Finland

From a wider perspective, dioxins in Baltic herring and salmon are only one of the many environmental health hazards (Fig. 9.). For example, they pose a smaller risk than methylmercury which is also present in fish, and their risk is not even close to the largest health risks originating from air pollution (which may be up to tens of thousands DALY in Finland alone).

It is possible that we have been somewhat overoptimistic about the current sperm concentrations, as reduction from subfertile background levels could increase the probability of infertility more than predicted in our model. Therefore, we conducted a sensitivity analysis on men with decreased sperm concentrations from an unrelated reason, which dioxins would be likely to reduce even further. For example, if the sperm concentration is 10 million/ml, the probability of infertility in 5 years is 0.32 based on the equation shown above. That increases to 0.4 at a dioxin concentration of 10 pg/g. If 10 % of the population had such low sperm concentration and if 20% of boys exceed 10 pg/g (as seems to be the case according to our model), then for example we would see in Finland 25,000 boys/year * 0.1 with low fertility * 0.2 with high dioxin * 0.08 absolute increase in infertility = 40 cases per year, each 2.5 DALY and thus 100 DALY in total. This is more than the 29 DALY estimated from the default model, but does not change the overall picture in Fig. 9. The individual risk per mother would be 0.1 and 0.03 mDALY/a per person, respectively (compare to Fig. 7). They are also much smaller than the 25,000 boys/year * 0.1 with low fertility * 0.32 absolute probability of infertility * 2.5 DALY = 2000 DALY due to infertility from all other causes of low sperm concentration in our sensitivity analysis.

IHME estimates the Finnish disease burden of male infertility of all causes at only 52 DALY/a; when the value includes female infertility, it is roughly doubled [44]. Thus, it seems that our estimates seem to overestimate rather than underestimate the sperm concentration problem.

Value of information was examined for specific decision scenarios, where a group of similar decisions were considered together.

Value of information was calculated for the total burden of disease in a random study country (Denmark, Estonia, Finland, and Sweden were not weighted by population), but using uncertainties for individual people. This approach ensures that the value of information is not underestimated, because at the population level, many uncertainties average out and are smaller than at the individual level.

The decision about recommending a small herring size has practically no expected value of perfect information (less than 1 DALY/a for a whole country) because switching from large to small herring is a no-regret option; in fact, in most cases, it is better than the other alternatives.

The decision about consumer policy (including improved information, better availability and usability of fish, and consumption recommendations) has an expected value of perfect information of 150 DALY/a, and there is some uncertainty about what should be recommended. The maximum net benefit is usually achieved by increasing Baltic fish intake. Therefore, the most important decision option which needs to be incorporated into the decision process is to increase factual information and fish availability (there would be expected loss of 1600 DALY/a if that option was not considered).

The analysis was also performed for the young female subgroup separately, assuming a situation where subgroup-specific policies would be both feasible and effective and would not affect the other subgroups. The expected value of perfect information was 190 DALY/a. At the same time, the total disease burden at stake is clearly smaller than with the whole population (see Fig. 7), because the disease burden from overall mortality and heart disease is small among young women. These two results together reveal that the uncertainties about what to recommend with respect to young women are clearly larger than with the other subgroups.

## Discussion

Dioxin and PCB concentrations have been constantly decreasing in Baltic fish for 40 years, and now they are mostly below the EU limits. In addition, the consumption of Baltic herring has been decreasing during the last decades and is now less than a kilogram per year, varying between age groups (older people tend to eat more), genders (males tend to eat more) and countries (Estonians tend to eat more and Danes less than the three other countries studied). People reported that better availability of easy-to-prepare products, interesting recipes, and reduced pollutant levels would increase their consumption of Baltic herring [11]. In contrast, recommendations to reduce consumption on average would have little effect.

According to this study, the health benefits of Baltic herring and salmon clearly outweigh health risks in people in age groups over 45 years. The benefits are similar to risks in the most vulnerable subgroup, women of childbearing age. The balance depends on value assumptions: risks prevail if exceeding the tolerable weekly intake as such (especially the new 2018 value) is given weight in the consideration. The analysis was robust in the sense that we did not find factual uncertainties that could significantly change the conclusions and would warrant postponing decisions in the hope of gathering new crucial information.

The largest health benefits originates from reduced all-cause mortality, coronary heart disease, breast cancer, and depression, and improved intelligence quotient of the child of a woman who consumes fish. Each of these effects has been linked to either fish in the diet or more specifically to omega-3 fatty acids, implying that a full palette of benefits is not available only from food supplements.

The risk of methylmercury to lower a child’s IQ appeared to be as large, or even larger, than all dioxin risks combined (Table 4). In fact, it is a common misconception that dioxins pose the largest risk if one eats Baltic fish. In addition, the dioxin-related cancer risk was not larger than the risk of developmental effects despite the fact that dioxins are reputed to be potent carcinogens.

There seems to be room for an updating of the risk communication about consumption of Baltic fish. This analysis suggests that dioxins are not the largest health risk related to these fish; and that the dioxin-related cancer risk is small in everyday life. The facts that dioxins are very potent per microgram of substance and that they cause cancer in laboratory animals after high doses [1] are irrelevant details in risk communication and instead, they provide a wrong impression of the actual risks to an individual’s health.

We found some no-regret policies. Promoting the consumption of small Baltic herring rather than large ones confers all of the health benefits but reduces the individual’s exposure to pollutants. Promoting the consumption of Baltic fish to population subgroups other than young females confers more health than harm. Finally, reducing dioxin emissions into the atmosphere will reduce concentrations not only in fish but also in dairy and meat products.

One critical question about this assessment is whether the beneficial effects are actually real and causal. The recent Cochrane review concluded that there is little if any cardiovascular benefit from omega-3 supplements [24]. Aung et al. conducted a meta-analysis of omega-3 supplement trials with more than 77,000 individuals [50]. They found only weak, border-marginal cardiovascular benefits and concluded that the analysis did not support the use of dietary omega-3 supplements. The US NCCIH has also commented on these studies: “Moderate evidence has emerged about the health benefits of eating seafood. The health benefits of omega-3 dietary supplements are unclear.” [51].

Indeed, omega-3 supplements may not affect diet composition, but each fish meal replaces another meal of some kind. These other dietary changes complicate estimates on the impacts of a specific nutrient. Therefore, it is warranted to look at epidemiological meta-analyses on fish consumption as a whole. Fish consumption has been reported to exert clear beneficial effects on all-cause mortality [27] and depression [28]. In contrast, marine omega-3 fatty acids, but not fish consumption, was linked to a reduced risk of breast cancer [26]. It seems plausible that not all of the effects are mediated specifically by omega-3 fatty acids, but that fish likely are sources of several beneficial nutrients and should be a part of healthy diet i.e. it is probably over-simplistic to claim that one compound is the source of all of the benefits.

Elizabeth Pennisi discussed several studies about the genetic variation of fatty acid metabolism and its links to cardiovascular risk [52]. The overall conclusion is that although these issues are not completely understood, there seems to be genetic variations in the health benefits of omega-3 fatty acids. Lauritzen et al. conducted a review on docosahexaenoic acid (DHA) and concluded that it was especially important for the developing brain during the fetal period and infancy, although there may be variation in intrinsic production and therefore in the need to obtain DHA from the diet [53]. This also implies that there can be complex variations within the population.

Irrespective of the actual cause, several beneficial effects from fish consumption make the case stronger: even if one endpoint turns out to be less important than previously thought, as has recently happened with omega-3 supplements and cardiovascular health, it is still unlikely that all of these results are false positives. In this assessment, we used the Cochrane best estimate of the omega-3 effect, which was described as “little, if any use for cardiovascular disease prevention”. Interestingly, the benefit was still larger than the risks associated with dioxin from the same fish. This result emphasizes the importance of quantitation and context: what is “small” depends on what it is being compared against.

A previous benefit-risk assessment was performed on omega-3 fatty acids and dioxins [54]. The study found scenarios where consumption of herring in the Netherlands would confer the benefits of omega-3 but dioxin exposure would remain below the tolerable intake at that time (14 pg/kg/week).

Another study concluded that Atlantic herring provided cardiovascular health benefits at consumption levels where the dioxin cancer risk remained acceptable [55]. A Chinese study also concluded that herring containing PCB7 concentrations 12.5 ng/g fresh weight could be used regularly and would confer health benefits without significant risks linked with the contaminants [56]. This is equivalent to approximately 2–3 pg/g fresh weight of TEQ, assuming that the PCB7/TEQ ratio is similar in Finnish and Chinese herring.

However, the National Food Agency of Sweden published a report which concluded that increased herring consumption would unnecessarily increase dioxin risks, since it would be possible to eat fish with less dioxins (e.g. smaller herring or other species) and still gain the same health benefits [57]. The report did not assess how consumption would change in practice, if Sweden abandoned its exemption to use large herring.

None of these benefit-risk assessments compared the magnitude of risks and benefits quantitatively by applying a common scale such as DALY. A more common approach has been to compare exposure estimates from a study to an administrative threshold. Clear conclusions can be drawn if the risks are below and the benefits above their respective thresholds, or vice versa. However, this approach is dubious in situations where there is a need for quantitative comparison of risk and benefits.

According to our experience, a threshold-type exposure-response function leads to a tendency to use the threshold as a strict guideline for action. This leads to inefficient allocation of resources, as it is unlikely that the risks would abruptly increase or benefits decrease.

In contrast, a quantitative benefit-risk assessment attempts to estimate the total benefits and total risks and then to compare these two values. In this approach, it is necessary to estimate all relevant endpoints, even if there is uncertainty about the mere existence of a causal effect. There is a reason for adopting this approach: if an uncertain endpoint is rejected from further scrutiny, mathematically it implies certainty about zero impact. Therefore, on the one hand, it is necessary to avoid omissions and try to produce a balanced quantitative view of both risks and benefits, and on the other hand, of their uncertainties.

This is also important for risk communication and risk perception. If all hazards are just “risky” or “not risky”, people fail to appreciate that some risks are worse than others. For example, our whole fish benefit-risk assessment shines an illustrative light when compared with other major environmental health risks: maybe it is not worth worrying too much if it draws attention away from more serious risks. Having said that, it should be remembered that even small risks are worth reducing if the costs of reduction are low or even negative.

The recent EFSA TWI recommendation for dioxins (2 pg/kg/week as compared with the previous TWI 14 pg/kg/week) dramatically increases the fraction of the population who are non-compliant in all four countries studied here. However, the implications of this policy decision are far from clear and require further discussion. We encourage both researchers and administrators to pay much more attention to comparing risks and benefits instead of only considering risks isolated from real, complex situations [58]. In fact, our results raise several questions.

First, the most sensitive outcome, namely the decrease in the sperm concentration, is only relevant for young women whose future children may be affected. In this respect, should the TWI be applied to all population subgroups?

Second, as dioxin exposure strongly relates to a diet consisting of otherwise healthy Baltic fish (especially Baltic herring) in the Nordic countries, should these health benefits be considered when changes to dioxin policy are announced? For example, the Swedish food safety authority did not raise this issue in their commentary about the new EFSA TWI [59].

Third, Baltic herring have also other important values in addition to their effects on health: there are the following factors that should not be ignored 1) economic (Baltic herring is the most abundant catch species by weight from the Baltic Sea), 2) ecological (sustainable yield of Baltic herring is large and the catch removes nutrients from the sea), 3) climate (Baltic herring could replace red meat and other climate-unfriendly food sources), 4) social (Baltic herring is an inexpensive local food), and 5) cultural (Baltic herring and salmon are an important part of coastal culture) [60, 61]. Moreover, Baltic herring has value for food security [5, 11, 62]. Should these values be considered when a dioxin policy is being devised? The BONUS GOHERR project found that all of these issues are considered important in our twenty-first century society [63, 64].

The results of the study should not be considered as exact magnitudes of the properties studied. We attempted to quantify real, measurable properties but acknowledge that these are just our best estimates of the actual truth, sometimes produced with only marginal data. We also tried to use probability distributions in a systematic manner to minimize any bias and also to take account of actual variations in populations. We had to make several assumptions e.g. about actual impacts of policies, how representative Finnish measurements are for fish in other countries, what background exposures to use, and how to derive disability weights or durations. Furthermore, we had to convert all outcomes into a single metric for policy and value-of-information analyses, and for this purpose, DALY seemed to be most feasible. We had to stretch the definition slightly to include non-disease outcomes, and we also had to use author judgement to estimate the impacts of competing risks, which are not directly observable from epidemiological data. Previous assessments have shown large health benefits related to fish, but since there is some uncertainty associated with these claims, we tried to be realistic but also tried to avoid underestimating risks, because that bias might weaken the arguments about safety of fish.

We have made the data, code, and reasoning available on Opasnet to facilitate the work of potential critics to find mistakes and false interpretations and also offer a place to publish comments and criticism. A quantitative benefit-risk assessment can actually be seen as one step in an iterative and collaborative process, where the understanding improves as other researchers make their own assessments, resulting in updated evaluations for use in future studies. Quantitative benefit-risk assessments would offer material for realistic discussions about making informed compromises between risks and benefits.

This study was not designed to answer value-based questions but it was able to identify some interesting points. The value of information was low for the remaining scientific uncertainties about dioxin risks, and the critical questions are related to the value questions mentioned above. Of course, dioxin sources and concentrations vary in different parts of Europe as well as in other parts of the world, but because of biomagnification, fish are invariably a typical source of many persistent pollutants. Political discussions and deliberation are needed about risk-benefit comparisons. Scientific facts are crucial, but they are not the only crucial elements in that discussion.

## Conclusions

In conclusion, despite the new evidence and at odds with the new EFSA TWI recommendation, this study indicates that Baltic fish are still a safe and healthy food for most population subgroups in the Nordic countries. A distinct subgroup, namely young women planning to have children, is of special concern. The health benefits in this subgroup are smaller than in the older age groups, and also there are potential but small risks to a child that is exposed during pregnancy and breast feeding. Experts do not agree on conclusions about this subgroup, but the scientific uncertainties actually do not play a large role. In contrast, value judgements are crucial when designing policies related to the dioxin problem of Baltic fish. These questions should be carefully discussed and deliberated by decision makers, experts, citizens, fishermen/women, and other stakeholders.

## Data Availability

The whole benefit-risk assessment was performed online at http://en.opasnet.org/w/Goherr_assessment, and all details (including data, code, results, descriptions, and discussions) are openly available, except for the personal data from the consumer survey. The consumer survey data has been converted to and published as synthetic data, i.e. data that does not represent any real individuals but that has similar statistical properties as the actual data. The datasets generated and analysed during the current study, together with the other material mentioned above, are available at Open Science Framework research data repository by the Centre for Open Science. https://osf.io/brxpt/

## Abbreviations

CHD: Coronary heart disease
CI: Confidence interval
DALY: Disability-adjusted life year
DHA: Docosahexaenoic acid
ED50: Effective dose 50
EFSA: European Food Safety Authority
EPA: Eicosapentaenoic acid
ICES: International Council for the Exploration of the Sea
IHME: Institute for Health Metrics and Evaluation
IQ: Intelligence quotient
MeHg: methylmercury
TEF: Toxic equivalency factor (of dioxins)
TEQ: Toxic equivalency quantity (of dioxins)
TWI: Tolerable weekly intake
WHO: World Health Organisation
