# Correction to: Health effects of nutrients and environmental pollutants in Baltic herring and salmon: a quantitative benefit-risk assessment

**DOI:** 10.1186/s12889-020-08501-2

**Published:** 2020-03-25

**Authors:** Jouni T. Tuomisto, Arja Asikainen, Päivi Meriläinen, Päivi Haapasaari

**Affiliations:** 1 Finnish Institute for Health and Welfare, Kuopio, Finland; 2 University of Helsinki, Helsinki, Finland

**Correction to: BMC Public Health (2020) 20:64**

**https://doi.org/10.1186/s12889-019-8094-1**

It was highlighted that the original article [1] contained a formatting error in the equations. These were all erroneously captured as Eq. 1. This Correction article shows the correct Eqs. 1–4. The original article has been updated.

Equation 1 (infant’s exposure)

1 $$ {C}_{s,i}=\frac{I_{a,m}\ast {t}_{1/2,m}\ast {f}_m\ast FE}{\mathit{\ln}2\ast B{F}_i}, $$

Equation 2 (probability of not getting pregnant)

2 $$ P(a)={\left(1-0.65\left(1+\frac{-0.39c}{c+10 pg/g}\right)\right)}^{2a} $$

Equation 3 (disease burden)

3 $$ Bo{D}_i= BoD\ast PA{F}_i= BoD\ast {f}_i\frac{R{R}_i-1}{f_i\left(R{R}_i-1\right)+1}, or $$

4 $$ Bo{D}_i={N}_i\ast L\ast Dw=P\ast {f}_i\ast U{R}_i\ast {E}_i\ast L\ast Dw $$

Equation 4 (value of information)

5 $$ VOI=E\left( ma{x}_i\left(U\left({d}_i\right)\right)\right)- ma{x}_i\left(E\left(U\left({d}_i\right)\right)\right), $$
